# Peering into the team role kaleidoscope: the interplay of personal characteristics and verbal interactions in collaborative problem solving

**DOI:** 10.3389/fpsyg.2024.1345892

**Published:** 2024-09-16

**Authors:** Siem Buseyne, Amelie Vrijdags, Sameh Said-Metwaly, Thierry Danquigny, Jean Heutte, Fien Depaepe, Annelies Raes

**Affiliations:** ^1^KU Leuven, Faculty of Psychology and Educational Sciences, Centre for Instructional Psychology & Technology, Leuven, Belgium; ^2^KU Leuven, imec research group itec, Leuven, Belgium; ^3^CIREL, Centre Interuniversitaire de Recherche en Education de Lille (ULR 4354), ULille, Lille, France; ^4^Hudson Belgium, Brussels, Belgium; ^5^Faculty of Education, Damanhour University, Damanhour, Egypt

**Keywords:** personality, team role behavior, verbal interaction, audio data, learning analytics, collaborative problem solving

## Abstract

The objective of this study is to explore the relationship between personality and peer-rated team role behavior on the one hand and team role behavior and verbal behavior on the other hand. To achieve this, different data types were collected in fifteen professional teams of four members (*N* = 60) from various private and public organizations in Flanders, Belgium. Participants’ personalities were assessed using a workplace-contextualized personality questionnaire based on the Big Five, including domains and facets. Typical team role behavior was assessed by the team members using the Team Role Experience and Orientation peer rating system. Verbal interactions of nine of the teams (*n* = 36) were recorded in an educational lab setting, where participants performed several collaborative problem-solving tasks as part of a training. To process these audio data, a coding scheme for collaborative problem solving and linguistic inquiry and word count were used. We identified robust links and logical correlation patterns between personality traits and typical team role behaviors, complementing prior research that only focused on self-reported team behavior. For instance, a relatively strong correlation was found between Altruism and the Team builder role. Next, the study reveals that role taking within teams is associated with specific verbal interaction patterns. For example, members identified as Organizers were more engaged in responding to others’ ideas and monitoring execution.

## 1 Introduction

Collaborative problem-solving (CPS) competencies are increasingly vital for enhancing efficiency, effectiveness, and innovation in contemporary society (Graesser et al., 2018; Neubert et al., 2015). CPS is “a process whereby two or more agents attempt to solve a problem by sharing the understanding and effort required to come to a solution and pooling their knowledge, skills and efforts to reach that solution” (OECD, 2017, p. 47). Despite the crucial role of CPS competencies in the job market, research conducted by organizations such as the OECD (2017) reveals that learners are often inadequately prepared for future job requirements demanding these competencies.

To effectively foster CPS competencies through training, a deep theoretical understanding of the underlying processes and the complex factors that influence them is needed (Fiore et al., 2018; Graesser et al., 2018; Macfarlane and Mayer, 2005). According to Graesser et al. (2018), team roles and personality traits could significantly influence CPS processes. However, these factors have yet to be documented empirically (Graesser et al., 2018).

Most research on team roles in collaborative learning (e.g., Raes et al., 2016; Pozzi, 2011; Schellens et al., 2007) has focused on the impact of assigning scripted roles to students on collaborative learning processes. Recent literature has also focused on roles that naturally emerge (e.g., Aranzabal et al., 2022; Marcos-García et al., 2015; Stahl et al., 2014). In general, and especially in the context of CPS, research on these emergent roles that are not pre-assigned needs further attention. Our study aims to address this gap by investigating the link between typical team role behavior and verbal interactions in CPS.

Role-taking can also be related to various personal characteristics, such as gender (Anderson and Sleap, 2004; Balderson and Broderick, 1996), job occupation (Balderson and Broderick, 1996), and personality traits (Davies and Kanaki, 2006; Marjanović et al., 2023). For instance, Davies and Kanaki (2006) showed that individuals with dominant interpersonal characteristics are more likely to take on roles involving organizing and coordinating tasks.

Most research on the link between personality traits and team roles has utilized Belbin’s (1993) team role structure (see Supplementary Appendix A). However, due to various shortcomings of this framework, Mathieu et al. (2015) developed the Team Role Experience and Orientation (TREO) framework (see Supplementary Appendix A). Research on the relationship between the TREO roles and personality traits is limited to Mathieu et al. (2015) study, which focused on self-reported team roles and involved participants from a limited number of contexts (i.e., military officers and business students). Therefore, this study aims to deepen the examination of the relationship between personality and typical team role behavior, evaluated by colleagues who are long-term collaborators with whom the participants have shared close working relationships, often spanning several years.

## 2 Theoretical framework

In what follows, we present the theoretical framework, organized as follows. The first section elaborates on the core concept of this study: team roles. This section introduces two foundational frameworks: the TREO framework (Mathieu et al., 2015) which will be used in this study, and Belbin’s (1993) model, which has been more frequently used in previous research. In the second section, the discussion centers on the interplay between team roles and personality traits. This discussion draws on prior research that utilizes the aforementioned team role frameworks and includes additional studies concerning team behavior and team effectiveness. The third section reviews previous research on the analysis of interactions among team members within the context of CPS. This section emphasizes the analysis of verbal interactions and outlines earlier investigations into the relationship between these verbal interactions and the concept of role-taking in collaborative contexts.

### 2.1 Team roles

Within teams, individual members assume various roles, each contributing unique strengths and capabilities to the group’s collective performance. Stewart et al. (2019) define a team role as a set of interrelated behaviors that an individual exhibits within a specific setting, especially during recurring interactions with others. These behaviors are not isolated actions but rather characteristic patterns of behaviors that individuals adopt in response to the demands of their environment and the dynamics of group interaction. Researchers have developed various taxonomies and frameworks to classify and understand the dimensions of role fulfillment (e.g., Barry, 1991; Belbin, 1993; Mumford et al., 2008).

#### 2.1.1 The Belbin team roles

Among the widely recognized frameworks, Belbin (2011) presents a notable one. This framework identifies nine distinct team roles; Resource investigator, Teamworker, Coordinator, Plant, Monitor evaluator, Specialist, Shaper, Implementer, and Completer finisher. Each role is associated with unique behavioral characteristics and strengths that individuals bring to a group setting. Descriptions of these roles are provided in Supplementary Appendix A.

Although Belbin’s framework has been extensively used in various organizational contexts, it has also faced criticism (Broucek and Randell, 1996). For instance, research by Aritzeta et al. (2007) suggests that the Team Role Self-Perception Inventory (Belbin, 2011) shows strong associations between some team roles, indicating weak discriminant validity among certain scales. Additionally, according to Mathieu et al. (2015), many other theories and frameworks for team roles (e.g., Barry, 1991; Mumford et al., 2006) lack comprehensive validation evidence.

#### 2.1.2 The team role experience and orientation framework

In response to this gap, Mathieu et al. (2015) synthesized the aforementioned theories and proposed the TREO framework. The TREO framework comprises six roles distributed across three categories: task-oriented, change-oriented, and socio-emotional. According to Gardner (2017), the study presented by Mathieu et al. (2015) provides evidence of discriminant validity for the TREO roles as measured by the TREO survey, affirming their distinctiveness from the Big Five personality domains. In the following paragraphs, we outline each of the TREO team roles and their documented connection with the Big Five personality domains, as identified by Mathieu et al. (2015) using a self-report survey measure of the TREO roles. An overview of the TREO team roles is provided in Supplementary Appendix A. This table also describes the hypothetical relationships between the Belbin and TREO team roles, based on Mathieu et al. (2015).

Within the task-oriented category of the TREO framework, two key roles are highlighted: the Organizer and the Doer. The Organizer takes on the responsibility of providing structure and direction to the group’s activities, taking on tasks such as observing, coordinating, and organizing (Griggs et al., 2021). Additionally, Organizers keep track of the group’s progress, ensuring that it aligns with established goals and timelines. Complementing the Organizer, the Doer takes on the tasks necessary for achieving group success, ensuring that deadlines are met and tangible outcomes are produced (Mathieu et al., 2015).

In the change-oriented category, the Challenger and Innovator roles help explore alternative perspectives and problem-solving approaches to avoid premature decision-making. The Challenger encourages the group to delve into different aspects of its assignment, often questioning the rationale behind decisions and ideas (Mathieu et al., 2015). This role involves behaviors such as asking “why” and critically evaluating the group members’ contributions (Griggs et al., 2021). Conversely, the Innovator generates novel knowledge, creative ideas, and innovative strategies to address challenges (Griggs et al., 2021; Mathieu et al., 2015).

The socio-emotional category includes the roles of Team builder and Connector, both of which focus on fostering a positive and collaborative group atmosphere. The Team builder plays a vital role in establishing group norms, facilitating decision-making processes, and maintaining a harmonious work environment (Mathieu et al., 2015). This role involves behaviors such as active listening, calming tense situations, and providing emotional support to team members (Griggs et al., 2021). On the other hand, the Connector is responsible for building and nurturing external relationships to ensure effective collaboration and a broader network of support (Mathieu et al., 2015).

Mathieu et al. (2015) explored the effectiveness of their self-report TREO predisposition measures in predicting peer-rated team role behaviors in group settings. Their findings indicate that the TREO self-report measure predicted peer ratings of role-related behaviors to some extent, with correlations ranging from.10 to.33 between self-ratings and peer ratings of the same TREO role.

### 2.2 The link between personality traits and team roles

According to Burch and Anderson (2009), the link between personality and teamwork is becoming increasingly prominent and needs further attention. To this end, previous research has been done studying the relationship between personality and self-report team role measures. Fisher et al. (2001) suggest that connections can be drawn between the Big Five domains of personality, and specific roles within Belbin’s framework. For example, Broucek and Randell (1996) have found moderate to strong positive correlations between the personality domain Conscientiousness and the team roles Implementer and Completer finisher. Similarly, research has looked into the relationship between the Big Five domains and the TREO survey. This exploration of the relationships between the TREO dimensions and the Big Five personality traits is an initial step in mapping the TREO’s nomological network (Gardner, 2017). Mathieu et al. (2015) investigated this relationship using a condensed version of the International Personality Item Pool scale (Donnellan et al., 2006). Following the correlation guidelines outlined by Gignac and Szodorai (2016), their research reports several significant and relatively strong correlations (i.e., equal to or higher than.30). Specifically, the Team builder demonstrated strong positive correlations with Agreeableness, Extraversion, and Conscientiousness. Both the Organizer and Doer role exhibited strong positive correlations with Conscientiousness and Extraversion. The Innovator role showed positive correlations with Extraversion and Openness. The Challenger role correlated relatively strongly with Openness and Extraversion, and the Connector role displayed the strongest relationship with Extraversion. To the best of our knowledge, Mathieu et al. (2015) is the only study reporting on the relationship between Big Five and the (self-reported) TREO roles. To date, no research has systematically explored the association between facets underlying the Big Five domains (i.e., the traits defining these domains, as exemplified by McCrae, 2020) and typical team role behaviors, as observed by peers.

### 2.3 Role-taking and verbal interactions in CPS

Beyond examining the link between personality traits and team roles, there is a need to investigate the link between these team roles and verbal interactions within collaborative environments. Particularly, in the context of CPS, researchers (e.g., Graesser et al., 2018) have emphasized the need for (semi-)automated assessments of CPS processes to facilitate both formative and summative feedback. As highlighted by Griggs et al. (2021), this involves gaining insights into team roles through observable indicators. Considering prior research on multimodal learning analytics in the context of CPS, various indicators can be used to evaluate this association. The following section provides an overview of these indicators, followed by a deeper exploration of the anticipated relationships between these indicators and team roles using the TREO framework.

#### 2.3.1 Verbal and non-verbal interactions in CPS

Given the advancements in technologies and techniques, including applications of artificial intelligence, research on the analysis of CPS interactions is evolving in multiple ways. These interactions among team members during CPS encompass both non-verbal and verbal aspects (Buseyne et al., 2023a). For example, through their Nonverbal Indexes of Students’ Physical Interactivity framework, Cukurova et al. (2018) describe how CPS can be assessed in students using video data. Praharaj et al. (2022) highlight various non-verbal indicators of collaboration quality, including pitch, intensity, total speaking time, interruptions, and speech overlap. For example, speaking time can be used as an indicator of the quantity of participation (Bachour et al., 2008; Terken and Sturm, 2010).

The quantity of participation can also be measured through verbal aspects of communication, including total word count and the number of utterances per team member (e.g., Buseyne et al., 2023a). Additionally, content analysis has been used to analyze the type of interactions in CPS (e.g., Stewart et al., 2019, 2021, 2023). In the following section, we will elaborate on this type of analysis.

#### 2.3.2 Content analysis using natural language processing

Content analysis is crucial for gaining deeper insights into the types of verbal interactions in CPS. During this process, team members’ utterances are annotated based on CPS-related categories (e.g., Sun et al., 2020), enabling researchers and observers to better understand the dynamics and effectiveness of CPS. Various schemes are used for coding CPS utterances. For example, Xu et al. (2024) used the PISA CPS framework to categorize chat interactions in an online environment. However, many of the frameworks, including the PISA CPS framework, are merely competency frameworks and were not specifically designed for coding CPS utterances. In contrast, the generalized competency model for CPS by Sun et al. (2020) contains specific indicators for the latent categories of CPS. Specifically, Sun et al. (2020) distinguish three main CPS facets: constructing shared knowledge, negotiation and coordination, and maintaining team function.

In the past, this annotation was mainly carried out manually. However, new natural language processing (NLP) techniques have emerged to automate the process. NLP, a subset of artificial intelligence, uses computational algorithms to process and interpret human language. Automatic speech recognition, a branch of NLP, enables faster transcriptions of team conversations. Additional NLP techniques ais in classifying utterances. For example, Stewart et al. (2021) used multiple techniques to build automated detectors for three critical facets of CPS: construction of shared knowledge, negotiation and coordination, and maintaining team function (Sun et al., 2020). According to Tan et al. (2022), such techniques can be adopted to support and assess collaborative processes, both for group outcomes and social interactions.

#### 2.3.3 Analysis of verbal interactions using linguistic inquiry and word count

In addition to analyzing verbal interactions at the utterance level, alternative methods exist for word-level analysis, such as linguistic inquiry and word count (LIWC), developed by Pennebaker et al. (2015a). LIWC analyses written or spoken language and categorizes it based on a predefined dictionary of linguistic and psychological categories. These categories include linguistic elements (e.g., pronouns, prepositions, articles), psychological attributes (i.e., emotions, cognitive processes), and summary statistics (e.g., words per sentence, word count).

Several LIWC categories are of interest for the analysis of CPS interactions. First, as mentioned earlier, word count and words per sentence are valuable for mapping the quantity of participation for an individual member. Second, personal pronouns (e.g., I, you, we) play an important role in CPS interactions. Research has shown that inclusive pronouns (e.g., we, us) contribute to a sense of group membership and cohesion within a team (Demmans Epp et al., 2017). Personal pronoun use is also linked to one’s orientation, whether self-oriented or collectively oriented, and to an individual’s status within the group (Boyd and Schwartz, 2021). Furthermore, the use of personal pronouns can reveal something about one’s status within the group. According to Tausczik and Pennebaker (2010), individuals with higher status more often make statements that involve others, while individuals with lower status tend to use more self-oriented language (i.e., first person personal pronouns). Third, negations (e.g., no, never) and interrogatives (e.g., how, what) may relate to specific linguistic behaviors of CPS team members. For example, negations may express opposition to an idea during negotiations (Stewart et al., 2019). Interrogative statements can indicate questioning actions, such as seeking clarification or challenging a proposed solution (e.g., Sun et al., 2020). Fourth, emotion words, including both positive (e.g., nice) and negative (e.g., hate) emotion words, are valuable sources of information in the context of CPS. Emotions provide insights into the cognitive, motivational, and relational aspects of CPS (Avry, 2021). Research indicates that LIWC effectively detects emotional expressions in language usage (Tausczik and Pennebaker, 2010). Fifth, cognitive process words (e.g., think, know, perhaps) and time words (e.g., end, until) are of interest for analyzing verbal interactions in CPS. Stewart et al. (2019) found that among the LIWC categories, certain cognitive process words, such as causations, insights, and differentiations, exhibit strong correlations with CPS processes associated with negotiation and coordination. Similarly, time-related words are associated with the category time management, referring to how team members deal with time limitations (Meier et al., 2007).

### 2.4 The link between role-taking and verbal interactions in CPS

Considering the aforementioned literature, several connections can be drawn between role-taking and verbal interactions in CPS. In what follows we give a brief overview of each team role based on the TREO framework.

Within the task-oriented category of the TREO framework, Organizers and Doers are primarily focused on task completion (Mathieu et al., 2015; Griggs et al., 2021). Previous research indicates that the behaviors of Organizers often involve setting goals, summarizing or clarifying team members’ contribution, and coordinating team actions (Griggs et al., 2021). These behaviors align with CPS categories such as responding to others’ ideas or proposed solutions, monitoring execution, time management, technical coordination, discussing strategies, and coordinating task division (Mathieu et al., 2015; Meier et al., 2007; Sun et al., 2020).

For the Doer, task completion is a prevalent behavior, and sharing knowledge and understanding of the task is essential for achieving completion (Belbin, 2011; Mumford et al., 2008). Therefore, Doers are expected to exhibit behaviors corresponding to sharing knowledge and understanding of problems and solutions (Griggs et al., 2021; Mathieu et al., 2015; OECD, 2017; Sun et al., 2020).

The change-oriented category, as described by Mathieu et al. (2015), encourages members to consider alternative approaches. The Challenger role encourages the team to explore all aspects of the task and consider alternative explanations and solutions (Griggs et al., 2021; Mathieu et al., 2015). This aligns with CPS behaviors such as establishing common ground (e.g., asking for further clarification, and giving feedback on the understanding of what the other is saying and asking questions). Challengers, who explore alternative solutions, are expected to use more interrogatives (e.g., why; Griggs et al., 2021).

The Innovator engages in generating new knowledge and strategies for task resolution (Mathieu et al., 2015). This aligns with CPS behaviors such as sharing knowledge and understanding of problems and solutions and discussing strategies (Sun et al., 2020).

Lastly, in the socio-emotional category, Team builders’ interpersonal processes are directed toward ensuring team success by integrating team members’ expertise and perspectives. This aligns with CPS behaviors such as coordinating task division and establishing common ground (Sun et al., 2020, 2022). Additionally, Team builder are expected to take initiatives to advance collaboration processes (Sun et al., 2020, 2022). Team builders, devoted to maintaining a positive work atmosphere, are expected to use more positive emotion words in their verbal interactions. Furthermore, Team builders’ interactions are expected to be more team-oriented, observable through the use of first-person plural pronouns.

### 2.5 Objectives and research questions

In summary, this study is based on two primary research objectives.

We observed that, to the best of our knowledge, research studying the relationship between TREO roles and the Big Five personality traits is limited to the study by Mathieu et al. (2015), which primarily focuses on results obtained in a military context and among business students. Further research is needed to gain additional insight into this relationship. This knowledge can enhance the formation, selection, training, and development of teams. Therefore, the primary objective of this study is to investigate the relationship between team members’ personality traits and their role-taking tendencies, using the peer rating system described by Mathieu et al. (2015). In line with this objective, the first research question (Q1) is formulated as follows: How does personality relate to typical team role behavior, as conceptualized through the TREO framework? Supplementary Appendix A includes the hypotheses related to this research question. These hypotheses are based on meta-analytic summaries of the correlations between TREO and the Big Five across four independent samples in Mathieu et al.’s (2014) study. In addition to the Big Five domains, this study includes personality facets to gain a more nuanced understanding.

Previous research (e.g., Griggs et al., 2021) has highlighted the need for more research on the relationship between role-taking and verbal interactions in different contexts. Therefore, our study’s second objective is to build upon existing research by exploring this relationship, especially within the CPS domain. The second, exploratory research question (Q2), is formulated as follows: How does a team member’s typical team role behavior, as conceptualized through the TREO framework, affect their verbal interactions in the context of CPS? Given the exploratory nature of this research question, no specific hypotheses are formulated. We employ innovative techniques to analyze verbal interactions, aiming to gain a better understanding of team role behavior.

## 3 Method

This study received ethical approval from the Ethical Committee of KU Leuven (G-2022-5202). Before participating, all participants received an information letter outlining the study’s objectives and data collection procedures. Additionally, participants completed an informed consent form. In the subsequent sections, we will provide a comprehensive overview of the study’s context and data collection procedures, followed by explanations of the data processing and data analysis methods employed.

### 3.1 Study context

This study was conducted as part of a larger project titled Supporting Teamwork in Ambient Learning Spaces. Within the scope of this project, a training program focusing on CPS was developed for workplace teams. Multiple training sessions were conducted in an educational laboratory setting, called the Edulab, located at KU Leuven in Kortrijk, Flanders, Belgium. This location was chosen because the Edulab is equipped with the necessary infrastructure and hardware for audio and video recordings. The CPS training program was structured into five phases. For a comprehensive overview of the training’s design, refer to Buseyne et al. (2023b). Data collection for this study occurred during the second phase of the training, which incorporated a CPS task enriched with several game elements.

### 3.2 Participants

#### 3.2.1 Full sample for examining the relationship between personality and team roles

Participants (*n* = 60; 35 males, 24 females, and 1 other) were recruited from 15 pre-existing teams, representing a mix of private and public organizations within the Flemish region. Participants had diverse backgrounds and held various job functions. In terms of age distribution, one participant was aged between 18 and 24, while 37% fell within the age range of 25 and 34, 43% between 35 and 44, and 12% between 45 and 54. Additionally, one participant fell within the age range of 55 to 64. Participants had an average tenure of 6.75 years (*SD* = 6.01) within their current organization. Their average duration of employment within the specific team involved in the study was 3.98 years (*SD* = 4.07).

#### 3.2.2 Selected sample for examining the relationship between role taking and verbal interaction

For the second research objective, we used a reduced sample of nine teams (36 participants, 21 males and 15 females) selected based on the availability and quality of the audio data. Among these participants, one was aged between 18 and 24, 36% were in the age group of 25 to 34, 39% between 35 and 44, and 19% were 45 and 54. One participant was aged between 55 and 64. The average duration of employment within their current organization was 8.5 years (*SD* = 6.97), while the average duration of employment within their current team was 4.60 years (*SD* = 4.95).

### 3.3 Data collection

In the days leading up to the start of the training, participants were completed two questionnaires. The first, the Business Attitudes Questionnaire (BAQ; Vrijdags et al., 2014) is a workplace-contextualized personality instrument certified by the British Psychological Society (2023). This questionnaire evaluates personality using the Big Five domains, each broken down into four specific sub-traits or facets. Additionally, the questionnaire assesses five compound traits, collectively categorized under the term “Professionalism.” The 25 BAQ facet scores are calculated by averaging the scores of six items per facet, while Big Five domain scores are calculated by averaging the scores of the related facets. A detailed overview of all BAQ facets and their grouping under the Big Five domains is provided in Table 1, while the interpretation of each facet is described in Supplementary Appendix A. The BAQ has been shown to correlate well with other personality inventories, both work-contextualized and non-contextualized, and to predict job performance (e.g., Wille et al., 2018). This suggests that the BAQ is a reliable and valid measure of personality traits relevant to the workplace.

**Table 1:** Overview of the different facets per domain of the Business Attitudes Questionnaire.

Emotional stability	Extraversion	Openness	Altruism	Conscientiousness	Professionalism
Relaxed	Leading	Abstract	People-oriented	Organized	Ambitious
Optimistic	Communicative	Innovative	Cooperating	Meticulous	Critical
Stress-resistant	Persuasive	Change-oriented	Helpful	Rational	Results-oriented
Decisive	Motivating	Open-minded	Socially Confident	Persevering	Strategic
					Autonomous

For a detailed description of each of the Business Attitudes Questionnaire facets, we refer the reader to Supplementary Appendix A.

In the second questionnaire, participants evaluated their colleagues’ team role behaviors, based on the six roles outlined in the TREO model (Mathieu et al., 2015; see Supplementary Appendix A). Participants used a five-point Likert scale to rate to what extent each team member typically exhibits behaviors associated with the six TREO roles during their everyday collaborative efforts. To facilitate this evaluation, participants received concise descriptions for each role. Additionally, they had the option to note instances where they felt unable to assess a specific role behavior for a team member. For example, for the Innovator role, participants rated behaviors such as: “someone who regularly generates new and creative ideas, strategies, and approaches for how the team can handle various situations and challenges. An Innovator often offers original and imaginative suggestions” (Mathieu et al., 2015, p. 16). Out of the 1,080 cases (i.e., three individual ratings for four team members across six roles), 91% were successfully coded. If at least two out of three members rated their colleague, the average score, across raters, for each role, was calculated for that particular member. If a colleague did not receive sufficient ratings, no score was utilized, resulting in missing data.

During the second phase of the training, participants engaged in an activity intentionally designed to stimulate CPS. The primary objective of this activity was to collectively solve as many problems as possible within a 30-min time limit. Each of these problems is referred to as a problem-solving interval. The problems spanned a range of abilities, including verbal, numerical, logical reasoning, spatial insight, detail orientation, and memory. Participants were not provided with specific instructions or guidelines on how to approach these tasks. Instead, each team had the autonomy to decide whether to assign specific roles to individual members during the task. Throughout the CPS activity, team-level conversations were recorded using individual headsets, overhead microphones, and computer microphones. Additionally, the process was video recorded using the video infrastructure of the educational lab.

### 3.4 Verbal data processing

For the second research objective, the audio data were transcribed manually by one of the researchers because automatic speech recognition software performed inadequately for the Flemish context. Participants’ utterances were annotated using a coding scheme for content analysis in computer-supported CPS (see Table 2 and Supplementary Appendix A), based on Meier et al. (2007) and Sun et al. (2020). An independent data coder received training on the coding scheme. To ensure coding reliability, both the appointed data coder and one of the authors independently coded the transcribed data from one of the nine teams. Differences between the coded utterances were discussed, and the coding scheme was refined to suit the specific context of this research. Inter-rater reliability testing was conducted for the coded utterances of one team, resulting in substantial agreement at both the overall item level (*κ* = 0.75) and the aggregated sub-category level (*κ* = 0.79) based on Cohen’s (1960) kappa (e.g., Landis and Koch, 1977). The remaining parts were coded by the appointed data coder. After completing the coding process, the relative frequency of each coding category (i.e., a percentage) was calculated per person and per task within the CPS activity. Specifically, per task, the relative frequency per problem-solving interval was determined by dividing the total number of utterances of a person for a specific coding category by the total number of utterances by that person.

**Table 2:** Overview of the categories and sub-categories of the coding scheme for analyzing verbal interactions during CPS.

Category	Sub-category
A: Establishing, constructing and maintaining shared knowledge and understanding	A1: Sharing knowledge and understanding of problems and solutions
	A2: Establishing common ground
B: Negotiating and coordinating for task completion and problem solving	B1: Responding to others’ ideas or proposed solutions
	B2: Monitoring execution
	B3: Time management
	B4: Technical coordination
	B5: Discussing strategies
C: Maintaining team function and organization	C1: Taking initiatives to advance collaboration processes
	C2: Coordinating task division

For a detailed overview of the coding scheme, we refer the reader to Supplementary Appendix A.

Next, LIWC (Pennebaker et al., 2015a,b) was used to assess affective, social and cognitive dimensions of participants’ interactions per problem-solving interval within the CPS task. The analysis reported in this article was performed using LIWC2015 with the Dutch dictionary (van Wissen and Boot, 2017). In our study, the LIWC output variables provide percentages of total words per person per problem-solving interval, similar to the relative frequencies of the CPS categories described earlier. For instance, a value of 10.1 for personal pronouns signifies that 10.1 percent of all the words used by a person in a specific problem-solving interval were personal pronouns. However, some measures are calculated differently; for example, utterance count, word count, and words per sentence represent absolute values.

### 3.5 Statistical analyses

Data analyses for this study were performed using R (version 4.3.1). For the analyses of Q1, regarding the relationship between personality traits and team roles, single-level correlation analyses were performed. The correlation coefficients were calculated using the stats package (version 3.6.2) and the corresponding *p*-values were calculated using the psych package (version 2.3.6). Corrected *p*-values are reported to control for the family-wise error (i.e., multiple hypothesis testing) using Bonferroni correction (Onwuegbuzie and Daniel, 1999).

Next, for Q2, i.e., to assess the relationship between role taking (i.e., independent variables) and verbal interactions (i.e., dependent variables), multiple multilevel linear regression models, using the restricted maximum likelihood procedure, were performed with the lme4 package (version 1.1–34). These models account for the fact that each dependent variable was measured multiple times per person by including the person as a level 2 variable. Additionally, group and task were included as covariates in all models. According to Leyland and Groenewegen (2020), with limited higher-level units, it is “better to perform a single-level analysis and include dummy variables for the higher-level units” (p. 38). Therefore, groups were not added as an extra level due to the limited number of groups. The Connector role was excluded from the Q2 analysis because the behaviors associated with this role, such as communicating with people outside the team, could not be observed in this study.

## 4 Results

### 4.1 Correlations between personality and typical team role behavior

Descriptive statistics for each of the personality domains, facets, and team roles are presented in Supplementary Appendix B. In what follows, we present the results of correlations between the TREO roles and the domains and facets of the BAQ. Only relatively large correlations with the BAQ domains are presented in the text. For a full overview of all correlation results, including non-significant correlations and correlations with the BAQ facets, refer to Table 3. Following Funder and Ozer (2019) and Gignac and Szodorai (2016), correlations of.30 or higher were considered relatively large. A simplified overview of these relationships is presented in Supplementary Appendix C.

**Table 3:** Correlations between the BAQ (domains and facets) and the TREO roles.

	Task-Oriented		Change-Oriented		Socio-Emotional	
	Organizer	Doer	Challenger	Innovator	Team builder	Connector
**Emotionals stability**	**−0.01**	**0.01**	**0.12**	**0.08**	**0.18**	0.27*
Relaxed	−0.21	−0.16	−0.05	−0.04	0.03	0.08
Optimistic	−0.04	0.13	0.03	0	0.17	0.20
Stress resistant	0.05	−0.05	0.15	0.07	0.18	0.24
Decisive	0.21	0.12	0.28*	0.24	0.15	0.28*
**Extraversion**	0.45***	**0.21**	0.38**	**0.22**	0.42**	0.49***
Leading	0.50***	0.16	0.40**	0.26	0.48***	0.49***
Communicative	0.38**	0.07	0.36**	0.20	0.21	0.32*
Persuasive	0.34**	0.26	0.33*	0.24	0.37**	0.38**
Motivating	0.30*	0.23	0.15	0.02	0.39**	0.44***
**Openness**	**−0.11**	**−0.03**	0.41**	0.41**	**−0.03**	**0.19**
Abstract	−0.13	0.04	0.28*	0.32*	−0.13	−0.03
Innovative	0.06	0.05	0.40**	0.41**	0.03	0.29*
Change oriented	−0.12	−0.15	0.30*	0.26	0.10	0.26
Open minded	−0.15	−0.08	0.35**	0.34**	−0.02	0.21
**Altruism**	0.40**	0.27*	**0.14**	**0.09**	0.41**	0.37**
People oriented	0.27*	0.18	0	−0.01	0.29*	0.20
Cooperating	0.47***	0.30*	0.16	0.16	0.51***	0.38**
Helpful	0.30*	0.08	0.13	0.03	0.17	0.20
Socially Confident	0.23	0.24	0.16	0.09	0.29*	0.34*
**Conscientiousness**	**−0.07**	**−0.09**	**−0.25**	−0.27*	**−0.19**	**−0.21**
Organized	0.07	0.11	−0.22	−0.25	0.08	0.02
Meticulous	−0.02	−0.09	−0.30*	−0.30*	−0.17	−0.20
Rational	−0.21	−0.27*	−0.03	−0.05	−0.37**	−0.31*
Persevering	−0.05	−0.01	−0.15	−0.12	−0.09	−0.13
**Professionalism**						
Ambitious	0.15	−0.01	0.20	0.04	0.11	0.15
Critical	−0.08	−0.10	0.34**	0.18	−0.18	−0.05
Result oriented	0.12	−0.02	0.27*	0.22	0.24	0.11
Strategic	0.07	−0.06	0.42***	0.23	0.04	0.14
Autonomous	0.05	−0.03	0.27*	0.16	−0.10	0

Personality domains are marked in bold. *p < 0.05, **p < 0.01, ***p < 0.001.

Regarding the Organizer, significant positive correlations were observed with two BAQ domains: Extraversion (*r* = 0.45) and Altruism (*r* = 0.40). These correlations were also relatively large for most of the facets related to these domains (i.e., Leading, Communicative, Persuasive, Motivating, People oriented, Cooperating, and Helpful). For the Doer role, a significant and relatively large correlation was found with the facet cooperating.

For the Challenger role, noteworthy positive correlations were observed with Extraversion (*r* = 0.38) and Openness (*r* = 0.41). Most of the correlations were significant and relatively large for the underlying facets (i.e., Leading, Communicative, Persuasive, Innovative, Change oriented, Open minded) and some of the Professionalism facets (i.e., Critical, Strategic). Additionally, significant correlations were found with the facets Decisive and Meticulous, which were positive and negative, respectively.

Regarding the Innovator role, a significant and relatively large correlation emerged with Openness (*r* = 0.41). Most of the correlations were also significant for the underlying facets (i.e., Abstract, Innovative, and Open minded). For Conscientiousness, a significant negative correlation was found with the Innovator role, though the coefficient was weaker (*r* = −0.27). The correlation with its facet Meticulous was relatively large and negative.

For the Team builder, strong positive correlations were found with Extraversion (*r* = 0.42) and Altruism (*r* = 0.41). For each of these domains, three of the underlying facets correlated significantly (i.e., Leading, Persuasive, Motivating, People-oriented, Cooperating, and Socially confident). An additional significant negative correlation was found with the facet Rational.

Lastly, the Connector role correlated significantly with Emotional stability (r = 0.27), Extraversion (r = 0.49), and Altruism (r = 0.37). For Emotional stability, only one significant correlation was found with the facet Decisive. For Extraversion, all facets correlated significantly (i.e., Leading, Communicative, Persuasive, and Motivating). Two out of four facets underlying the domain Altruism correlated significantly with the Connector role (i.e., Cooperating and Socially confident). Additionally, a significant negative correlation was found with the facet Rational.

### 4.2 Relationships between team role behavior and verbal behavior

Table 4 presents the results of the multilevel regression models examining the relationship between the TREO roles and the CPS categories. Table 5 presents the relationship between the TREO roles and the LIWC categories.

**Table 4:** Results of the multilevel regression analysis showing the relationship between TREO dimensions and the relative frequencies of the selected LIWC categories.

	A. knowledge & understanding	A1. sharing knowledge	A2. establishing common ground	B. negotiating & coordinating	B1. responding	B2. monitoring execution	B3. time management	B4. technical coordination	B5. Discussing strategies	C. team functioning	C1. advancing collaboration
(Intercept)	49.34 (4.66)***	21.43 (3.87)***	27.91 (3.61)***	43.78 (4.66)***	7.75 (1.88)***	5.81 (2.57)*	4.92 (1.50)**	11.46 (2.93)***	8.64 (2.75)**	1.77 (0.93)	6.86 (2.66)**
Organizer	−1.77 (1.61)	−1.77 (1.36)	0.02 (1.24)	1.63 (1.61)	1.39 (0.65)*	2.58 (0.89)**	−0.86 (0.59)	−0.68 (1.01)	−0.62 (0.95)	0.14 (0.35)	−0.78 (0.92)
Doer	3.38 (2.31)	−0.20 (1.94)	3.59 (1.78)*	−2.13 (2.30)	−0.17 (0.93)	0.16 (1.27)	−0.51 (0.82)	−1.20 (1.44)	−1.67 (1.36)	−0.35 (0.50)	−1.20 (1.31)
Challenger	1.58 (3.20)	−1.85 (2.69)	3.45 (2.47)	−3.82 (3.19)	−0.55 (1.28)	0.89 (1.76)	−1.09 (1.14)	−3.50 (2.00)	−0.37 (1.88)	0.34 (0.69)	−0.45 (1.82)
Innovator	1.97 (3.75)	5.48 (3.15)	−3.54 (2.89)	1.40 (3.73)	0.12 (1.51)	−3.15 (2.06)	0.68 (1.31)	4.21 (2.34)	0.22 (2.21)	−0.32 (0.80)	0.25 (2.13)
Team builder	−2.79 (1.95)	−3.00 (1.64)	0.21 (1.50)	−0.27 (1.94)	−0.19 (0.78)	−0.51 (1.07)	0.82 (0.71)	0.56 (1.22)	1.35 (1.15)	0.53 (0.43)	0.79 (1.11)
AIC	1958.59	1868.21	1850.11	1961.41	1567.01	1703.22	1368.68	1759.14	1733.03	1188.66	1717.94
BIC	2038.46	1948.07	1929.97	2041.27	1646.87	1783.08	1448.54	1839.00	1812.89	1268.52	1797.80
LL	−956.30	−911.10	−902.05	−957.70	−760.51	−828.61	−661.34	−856.57	−843.51	−571.33	−835.97
Between-person variance	0.94	2.93	0.00	0.00	0.00	0.00	2.82	0.00	0.00	0.84	0.00
Within-person variance	282.55	184.87	171.75	286.85	46.59	87.28	17.40	112.94	100.14	7.73	93.41

Regression coefficient estimates are shown, along with the standard errors and the indicator of the corresponding *p*-value. *p < 0.05, **p < 0.01, ***p < 0.001. A full overview of the categories (i.e., A, B, C) and sub-categories (i.e., A1, A2, etc.) is presented in Table 2.

**Table 5:** Results of the multilevel regression analysis showing the relationship between TREO dimensions and the relative frequencies of CPS categories.

	Words per sentence	Word count	I-pronoun	We-pronoun	Negations	Interrogatives	Positive emotion words	Negative emotion words	Cognitive process words	Time
Intercept	6.19 (0.42)***	218.04 (25.49)***	2.89 (0.80)***	3.94 (0.55)***	1.59 (0.68)*	0.77 (0.42)	0.82 (0.49)	0.05 (0.23)	15.57 (1.55)***	7.21 (0.94)***
Organizer	−0.18 (0.17)	−7.88 (9.82)	0.24 (0.29)	−0.40 (0.20)*	0.24 (0.23)	0.21 (0.15)	0.10 (0.18)	0.18 (0.08) *	−0.75 (0.58)	−0.45 (0.32)
Doer	−0.18 (0.23)	−28.84 (13.76)*	0.75 (0.41)	−0.18 (0.29)	−0.38 (0.33)	−0.15 (0.21)	−0.47 (0.25)	0.07 (0.11)	−0.77 (0.81)	−1.48 (0.46)**
Challenger	−0.27 (0.32)	−38.72 (19.11)*	0.33 (0.57)	−0.40 (0.40)	0.71 (0.46)	0.27 (0.29)	−0.54 (0.35)	0.29 (0.16)	−0.59 (1.13)	−1.19 (0.64)
Innovator	0.48 (0.37)	68.38 (22.03)**	−0.87 (0.67)	0.70 (0.46)	−0.60 (0.54)	−0.34 (0.34)	0.68 (0.41)	−0.24 (0.18)	1.13 (1.31)	2.31 (0.75)**
Team builder	−0.42 (0.20)*	−4.73 (11.82)	−0.15 (0.35)	0.40 (0.25)	0.25 (0.28)	0.13 (0.18)	0.15 (0.21)	0.11 (0.09)	0.31 (0.69)	0.65 (0.39)
AIC	792.26	2619.23	1166.73	992.68	1124.43	896.82	968.78	650.95	1436.19	1267.48
BIC	872.13	2699.09	1246.59	1072.54	1204.29	976.68	1048.64	730.81	1516.05	1347.34
LL	−373.13	−1286.62	−560.36	−473.34	−539.22	−425.41	−461.39	−302.47	−695.09	−610.74
Between-person variance	0.25	689.07	0.30	0.19	0.00	0.04	0.07	0.00	1.65	0.00
Within-person variance	1.21	5609.95	7.19	3.20	6.06	2.10	2.91	0.68	24.60	11.72

Regression coefficient estimates are shown, along with the corresponding standard error in parentheses and the *p*-value. *p < 0.05, **p < 0.01, ***p < 0.001.

First, for the role of Organizer, significant positive relationships were found with the sub-categories “responding to others’ ideas or proposed solutions” and “monitoring execution.” In terms of linguistic inquiry, a significant negative relationship was observed with the category We-pronoun and a positive relationship was found with the word category Negative emotions.

Second, for the Doer, a significant positive relationship was found with “establishing common ground.” Additionally, significant negative relationships were observed with Word count and Time.

Third, for the Challenger role, no significant relationships were found with the CPS categories. However, a significant negative relationship was observed with Word count in terms of word use.

Fourth, no significant relationships were found between the Innovator role and the CPS categories. However, significant positive relationships were found with Word count and the LIWC category Time.

Lastly, for the Team builder role, the observed relationships with CPS categories were not significant. In terms of LIWC, a significant negative relationship was found with Words per sentence.

## 5 Discussion and concluding remarks

### 5.1 Unravelling the link between personality and team roles

The first objective of this study was to explore the relationships between personality traits and typical team role behavior as observed and rated by close, long-term collaborators. Building on prior research (i.e., Mathieu et al., 2015), the current study provides additional insights into how personality characteristics may influence one’s predisposition to take up specific roles within a team. A summarized overview of the findings is presented in Supplementary Appendix C.

Various relationships identified in this study align with research by Mathieu et al. (2015), who studied links between the Big Five and TREO team roles using a self-report questionnaire. Particularly, Organizers displayed relatively strong positive correlations with Extraversion, Challengers and Innovators had strong correlations with Openness, the roles of Team builder and Connector positively correlated with Extraversion, and Team builders correlated positively with Altruism. Additionally, our study revealed numerous interesting connections that were not yet revealed in Mathieu et al. (2015) research. Particularly, our work includes a deeper, facet-level examination of personality, and the relationship with observed, peer-rated team role behavior. The following section provides a thorough interpretation of the identified patterns.

An individual assuming the role of Organizer within a team, is characterized by their ability to establish structural frameworks for the team’s activities, delineating the essential tasks to be executed (Mathieu et al., 2015). Consequently, our study shows that Organizer behavior exhibits a significant correlation with Leading, a personality facet encompassing leadership qualities, delegation proficiency, and instructional competence. Next, individuals perceived by their team members as adopting the organizer role also tend to demonstrate higher scores on Communicative, indicating their proclivity for extensive and articulate verbal interactions, as well as Persuasive, suggesting their perceived effectiveness in persuading others of their ideas and viewpoints. In the context of structuring tasks and monitoring past activities, one might naturally assume a positive correlation between Organizer and Conscientiousness, and with the Organized facet in particular. However, our research did not confirm this assumption. The Organized facet primarily reflects the capacity to plan and structure one’s own work, with individuals excelling in this facet often displaying a degree of inflexibility. Contrarily, Organizer, within a team, entails taking the lead in structuring and shaping the group’s work, placing a strong emphasis on the social aspect of team collaboration.

Interestingly, we found that the Doer role exhibited no robust correlations with any of the personality domains and facets. However, one relationship of smaller magnitude was found with Cooperating. This suggests that the Doer can be described as the dependable individual who takes on tasks and may not possess distinct characteristics but excels as a reliable executor.

When examining the definition of Challenger role (Mathieu et al., 2015), several aspects emerge, that correspond with various facets of the BAQ. Encouraging and motivating the team aligns with Leading. The disposition to consider alternative assumptions, explanations, and solutions, is associated with Open-minded and Innovative. Consistently, questioning and offering constructive criticism mirrors the facet Critical. Additionally, engaging in debates resonates with Communicative. All these BAQ facets exhibit strong, positive correlations with being perceived as a Challenger by team members. For this role, the findings underscore the link between individual’s self-perceptions of their personality and how they are perceived by their colleagues. Intriguingly, Strategic also demonstrates a strong positive correlation with the Challenger role. This connection may be attributed to the underlying motivations for assuming this role. Strategic signifies a heightened emphasis on long-term planning and adopting a more expansive viewpoint, as opposed to fixating solely on immediate tasks and ongoing projects. Individuals possessing a stronger proclivity for long-term orientation are likely to display a greater inclination toward challenging the status quo, thus assuming the challenger role.

The definition of the Innovator role shares certain similarities with that of the Challenger role, albeit within more constrained boundaries. While a Challenger encourages the team to explore alternative and innovative explanations and solutions, an Innovator primarily stands out as an individual who consistently generates fresh, creative, and original ideas independently. This role displays the most robust correlations with Innovative and Open-minded. Nevertheless, what is noteworthy is the extent to which these dimensions of an individual’s self-perception are accurately perceived by their colleagues during team collaboration.

A Team builder actively contributes to setting group norms and fostering a positive work environment (Mathieu et al., 2015). This aligns with the positive correlation observed with Leading. Individuals scoring high on Leading are more inclined, in comparison to those with lower scores on this facet, to take on a prominent role within the team. This may involve activities like establishing norms and fostering a positive work environment that garners attention and respect from their peers. The Team builder role also entails the ability to motivate team members, a characteristic substantiated by the strong correlation with Motivating. Furthermore, the Team builder role exhibits a significant positive correlation with Persuasive, indicating that these individuals perceive themselves as skilled at articulating and convincing others of their ideas. To attain the esteemed status of Team builder, one must project themselves as a genuine ‘people person’. Conversely, a negative relationship was observed with Rational. Team members who mostly base their decisions on empirical data, prioritize rational arguments, and exercise discernment in their interactions, as prescribed by Rational, may not typically be seen by their team mates as dedicated to fostering a positive work atmosphere or offering support, as is the case with the Team builder.

The correlations with the BAQ facets exhibit remarkable similarity between the Connector and Team builder roles. However, the distinction lies in that the Connector role involves establishing connections with stakeholders beyond the team, rather than focusing on fostering relationships within the team. Consequently, those who take on the role of connectors tend to excel as adept networkers. Notably, among all team roles, the Connector displays the strongest correlation with Socially Confident, emphasizes characteristics such as amiability, spontaneity, and proficiency in networking.

### 5.2 Unravelling the link between team roles and verbal behavior

As a second research objective, our study investigated the relationship between typical team role behavior rated by colleagues, and verbal behavior, including the relative frequencies of types of utterances and LIWC measures, during CPS.

Team members identified as Organizers exhibited a higher relative frequency of utterances related to “responding to others’ ideas or proposed solutions” and “monitoring execution.” These findings align with previous research by Griggs et al. (2021), which suggests that Organizers often engage in coordinating team actions, summarizing team members’ contributions, and setting goals. Additionally, team members rated as Organizers incorporated fewer we-pronouns, which may be an indication of a less collective orientation in verbal interaction. Furthermore, this role was associated with a higher use of negative emotion words. This particular finding warrants further exploration.

According to Griggs et al. (2021), the role of Doer is generally associated with supporting group memory, task execution, and maintaining task focus. The significant higher involvement of Doers in establishing common ground identified in the current study aligns with this description. By fostering an environment where information is clearly communicated and understood by the group, Doers enhance their ability to execute tasks effectively and maintain focus. Additionally, we observed that Doers had a lower word count and exhibited a less frequent use of time-related words in their conversations during CPS tasks. This may suggest that Doers prioritize a more action-oriented approach over engaging in extended team interactions. Further research is needed to verify this explorative finding.

Similarly to the Doer, team members identified as Challengers had a lower word count, indicative of less verbal interaction. Aligning with Griggs et al. (2021), this could be attributed to their focus on concise, targeted contributions aimed at questioning and critiquing ideas rather than engaging in extended dialogues. However, this concise communication style was not significantly observed in terms of average words per sentence.

Griggs et al. (2021) demonstrated that Innovators engage in behaviors such as generating ideas, sharing information, and working independently. Furthermore, we observed that individuals embodying the Innovator role had a higher word count and used more time-related words. This higher verbal interaction could be attributed to the nature of their activities, which involve extensive idea generation and detailed information sharing. However, the increased use of time-related words does not align directly with the typical role description of Innovators, suggesting that further exploration is needed.

Lastly, the Team Builder role, which typically involves managing conflicts, fostering a positive environment, and supporting consensus (Griggs et al., 2021; Mathieu et al., 2015), was associated in our study with a lower number of words per sentence (i.e., shorter sentences). This communication style may align with the nature of their role, which generally focuses on building consensus and maintaining a harmonious team environment instead of engaging in argumentation processes, which requires longer sentences.

### 5.3 Limitations of the current study

It is important to acknowledge certain limitations of this study. First, this study relies on intrinsically subjective self-report personality measures and peer ratings for the evaluation of typical team role behaviors, as well as non-automated human coding for content analysis. Although validated instruments were used, and inter-rater reliability was ensured, this could still introduce biases.

Second, the study was conducted within a specific context and with a limited sample size, which may affect the generalizability of the findings. Additionally, participants were not required to rate all their team members, resulting in some team members not receiving a full set of ratings for certain roles. However, this flexibility was necessary to ensure the validity of the ratings provided.

Third, it is important to note that the roles were assessed before the participants engaged in the CPS tasks. Therefore, the role evaluations were based on general impressions and long-term collaboration rather than the specific CPS tasks performed during the study. This approach was chosen to minimize the potential influence of the specific task characteristics on the role ratings. Nevertheless, this might have affected the findings.

Fourth, this study did not consider the team compositions and the distribution of personality traits within teams. Instead, we controlled for the overall group effect in our regression analyses to account for any potential influence of team characteristics.

Fifth, since team members had been working together for a longer time and the tasks they needed to perform for our research study were rather short, we could not assess how the verbal behavior in relation to the roles evolved over time.

### 5.4 Strengths of the current study

As stated in the title of our study, we aimed to peer into the kaleidoscopic view of team roles in the context of CPS, which is known as a complex context to investigate, by incorporating various measures and frameworks. Our research on the relationship between (a) personality domains and team role behaviors and (b) team role behaviors and verbal interactions is distinct from previous research in several ways.

To assess the relationship between personality domains and team role behavior, our study integrates additional facets labelled under Professionalism, alongside the traditional Big Five facets, providing a more comprehensive exploration. The simplicity of the Big Five serves as a valuable tool for establishing a shared vocabulary among researchers. However, it is increasingly accepted that a greater number of personality constructs offer significant benefits for both theoretical and practical applications (e.g., Stanek and Ones, 2018).

Additionally, we provide an overview of the correlations not only for each role and personality domain but also on the level of the personality facets, offering a more in-depth analysis. Merely making a conceptual distinction between facets within the Big Five domains is insufficient unless it holds empirical significance. When looking at our results, focusing on the relationships between the facets and domains of the BAQ and the TREO team roles, it proves useful to delve into the different facets of the BAQ, rather than solely concentrating on the broader Big Five domains. For instance, when considering the Conscientiousness domain, it appears unrelated to the Team builder role. Nevertheless, at the facet-level, a robust negative correlation emerges with the Rational facet. Evidently, team members who base their decisions on facts and figures, prioritize rational arguments, and exercise discretion in their interactions are not commonly perceived as Team builders. Such individuals may not typically invest effort in fostering a positive working atmosphere within the team, offering solace to stressed teammates, or providing motivation to those facing challenges. This principle also operates the other way around, as altruism displays a robust correlation with both being perceived as a Team builder and a Connector. When considering the distinct facets comprising the Altruism domain, neither of these team roles correlates significantly with the Helpful facet. Hence, it becomes apparent that demonstrating consideration and willingness to assist and help others does not contribute significantly to Team builder or Connector behaviors, as perceived by colleagues. The domain-level correlation predominantly arises from the strong positive association with the Cooperating facet, which primarily reflects a propensity to involve others and prefer teamwork over solitary work, rather than a focus on being helpful. The Helpful and Cooperating facets represent two distinct aspects within Altruism, both of conceptual and empirical importance in the context of team collaboration, as shown by our research.

Another strength, compared to similar research, is that our participants occupied diverse job roles across various sectors and were drawn from authentic teams characterized by longstanding collaborations. Despite the diverse nature of their job roles and sectors, we identified strong correlations, emphasizing the relevance of our findings across a broad spectrum of real-world team dynamics.

To examine the relationship between team role behavior and CPS processes, our study employed innovative methods not previously utilized for this purpose. Specifically, we used measures obtained through (a) content analysis, based on a CPS coding scheme and (b) LIWC. The identification of types of CPS utterances associated with each role enriches our comprehension of team dynamics. Our study, for instance, showed that organizers exhibited a higher relative frequency of utterances related to responding to others’ ideas or proposed solutions and monitoring execution. This aligns with prior research by Griggs et al. (2021). Second, the integration of LIWC analysis provided an additional layer of insight into CPS processes. Whereas the coded CPS categories solely focus on what is being communicated, LIWC provides insights into the nuances of how communication unfolds such as the amount of communication (i.e., word count). However, it is important to recognize that the correlations observed between specific verbal behaviors and team roles are exploratory and descriptive.

### 5.5 Implications for team-learning in professional contexts

The outcomes of this study hold significant implications for understanding the interplay between personality self-perceptions, peer-rated team role behavior, and verbal behaviors in the context of computer-supported CPS.

First, our findings underscore the importance of considering individual self-perceptions of personality as critical drivers of team role behaviors. Hogan and Roberts (2000) posit that personality self-perceptions shape individuals’ identities, interactions, and the roles they are willing to undertake. The convergence with other-ratings of team role behaviors highlights the robustness of these self-perceptions of personality. Understanding how individuals perceive their own personalities contributes to unravelling the dynamics of role allocation within teams. The successful demonstration that self-report personality measurements not only predict individual behavior but also correlate with typical team role behavior, provides a bridge between individual and team-level assessments. This suggests that personality assessments can offer valuable insights into team dynamics, aiding coaches and trainers in developing teams and their members. Furthermore, the robust correlation between individual self-perceptions of personality and how individuals are perceived by others in a team context adds validity to personality assessments, emphasizing their utility in predicting not only individual but also team-related behaviors.

Second, the study provides a unique contribution by demonstrating the applicability of peer-rated team role measurements for predicting individuals’ behavior in teams. Employers, coaches and trainers seeking to understand how candidates or employees are likely to behave within a team context can leverage these insights.

Third, as shown by Mathieu et al. (2015), self-report team role questionnaires often exhibit rather low correlations with peer-rated team role behavior. This raises doubts about the effectiveness of self-report team role questionnaires as reliable predictors. Our study suggests that personality assessments provide an alternative indicator for typical team role behavior instead of self-report team role questionnaires. Future research could compare the predictive ability of self-reported team role questionnaires compared to personality assessments for typical team role behavior.

### 5.6 Future research opportunities

This research opens avenues for further exploration of the behaviors associated with specific team roles in various contexts. Future research could also examine the relationship between team roles and both individual and team performance measures. In addition to the methods used in this study, researchers should explore additional methods for analyzing both verbal-and non-verbal behaviors. This includes using additional variables, such as analytical thinking and clout, available in newer versions of LIWC (Pennebaker et al., 2022), which were not accessible in the Dutch language at the time of this study.

To address the aforementioned limitations, future research should expand the sample size and include diverse contexts to enhance the generalizability of the findings. A larger sample size would allow the inclusion of the team as a higher-order structure in hierarchal regression analysis. Additionally, this would enable the exploration of other analysis methods, such as latent profile analysis.

Future studies should also consider examining the impact of different assessment timings on role evaluations and task performance. Investigating whether assessing team roles before or after specific tasks yields different insights and could help to refine our understanding of role dynamics in collaborative settings.

Moreover, future research could explore the causal effect of team composition and the distribution of personality traits within teams on verbal and non-verbal interactions during CPS. Understanding how the collective personality profile of a team affects its functioning, and effectiveness could offer deeper insights into team dynamics.

Furthermore, previous research has examined the associations between personality traits and team performance in a curvilinear manner. For instance, Curşeu et al. (2019) identified an inverted U-shaped association between Openness and contributions to teamwork in some studied samples. Therefore, future research could explore the relationships studied here using a curvilinear approach.

## Data Availability

The original contributions presented in the study are included in the article/Supplementary material, further inquiries can be directed to the corresponding author.
