# Corrigendum: Peering into the team role kaleidoscope: the interplay of personal characteristics and verbal interactions in collaborative problem solving

**DOI:** 10.3389/fpsyg.2024.1538778

**Published:** 2024-12-18

**Authors:** Siem Buseyne, Amelie Vrijdags, Sameh Said-Metwaly, Thierry Danquigny, Jean Heutte, Fien Depaepe, Annelies Raes

**Affiliations:** ^1^KU Leuven, Faculty of Psychology and Educational Sciences, Centre for Instructional Psychology & Technology, Leuven, Belgium; ^2^KU Leuven, imec research group itec, Leuven, Belgium; ^3^CIREL, Centre Interuniversitaire de Recherche en Education de Lille (ULR 4354), ULille, Lille, France; ^4^Hudson Belgium, Brussels, Belgium; ^5^Faculty of Education, Damanhour University, Damanhour, Egypt

**Keywords:** personality, team role behavior, verbal interaction, audio data, learning analytics, collaborative problem solving

In the published article, there was an error in the legends for **Tables 4**, **5** as published. The words “after stepwise deletion” were included incorrectly. The corrected legends appear below.

**Table 4** Results of the multilevel regression analysis showing the relationship between TREO dimensions and the relative frequencies of CPS categories.

**Table 5** Results of the multilevel regression analysis showing the relationship between TREO dimensions and the relative frequencies of the selected LIWC categories.

The authors apologize for this error and state that this does not change the scientific conclusions of the article in any way. The original article has been updated.

