# Supplementary material for: Peering into the team role kaleidoscope: the interplay of personal characteristics and verbal interactions in collaborative problem solving
Source: Front Psychol. 2024 Sep 16;15:1345892. doi: 10.3389/fpsyg.2024.1345892 (PMC11440957; doi:10.3389/fpsyg.2024.1345892)
Supplement: Supplementary file 1 [file Data_Sheet_1.docx]

# Appendix A

This appendix includes an overview of the Belbin team roles (see Table A1), an overview of TREO team roles (see Table A2), an overview of the hypothesis of Q1 (see Table A3) descriptions of the BAQ facets (see Table A4), as well as a detailed overview of the coding scheme that was used in this study (see Table A5).

**Table A1**

Overview and Description of the Belbin Team Roles

| **Role** | **Description** |
| --- | --- |
| Completer finisher | Completer finishers spot errors and omissions that others might overlook, ensuring that important details are not forgotten. |
| Coordinator | Coordinators are confident and stable team members who can clarify goals, promote decision-making, and delegate tasks effectively. |
| Implementer | Implementers are practical and reliable, translating team concepts and plans into a working brief and carrying it out efficiently. |
| Monitor evaluator | Monitor evaluators are capable of evaluating ideas for their viability, making them crucial in decision-making processes. |
| Plant | Plants are the main source of innovation and ideas, often providing unique solutions to complex problems. |
| Resource investigator | Resource investigators develop contacts on behalf of the team and explore external resources. |
| Shaper | Shapers are dynamic and driven, challenging the team to improve and excel and driving the team to achieve its goals. |
| Specialist | Specialists bring a certain set of specialized skills or knowledge to the table. |
| Teamworker | Teamworkers support others and encourage harmony and unity within the team. |

*Note*. The content in this table is based on Belbin (1993, 2011), Anderson and Sleap (2004), Aranzabel et al. (2022), and Aritzeta et al. (2007).

**Table A2**

Overview and Description of TREO Team Roles, Corresponding Higher-Order Constructs, and Related Belbin Team Roles

| **Higher order construct** | **Role** | **Definition** | **Related Belbin team roles** |
| --- | --- | --- | --- |
| Task-oriented | Organizer | An individual who organizes the team's activities, monitors their achievements, and tracks their progress against goals and deadlines. | Coordinator, Shaper |
|  | Doer | A person who readily accepts tasks and ensures their completion. A "Doer" is reliable for meeting deadlines and taking on responsibilities to guarantee the team's success. | Completer finisher, Specialist, Implementer |
| Change-oriented | Challenger | An individual who encourages the team to thoroughly investigate all aspects of a situation and consider different assumptions, explanations, and solutions. A Challenger frequently asks "why" and is at ease with critiquing and debating. | Monitor Evaluator |
|  | Innovator | An individual who consistently comes up with new and creative ideas, strategies, and approaches for the team to address various situations and challenges. An Innovator frequently provides original and imaginative suggestions. | Plant |
| Socio-emotional | Team Builder | An individual who helps set norms, supports decisions, and fosters a positive work environment within the team. A Team Builder soothes members when they are stressed and motivates them when they feel down. | Teamworker |
|  | Connector | An individual who facilitates connections between the team and external people, groups, or stakeholders. Connectors maintain positive relationships between the team and external parties. | Resource investigator |

*Note*. The content in this table is based on Mathieu et al. (2015).

**Table A3.**

Overview of the Hypotheses of Q1

|  | **Organizer** | **Doer** | **Challenger** | **Innovator** | **Team Builder** | **Connector** |
| --- | --- | --- | --- | --- | --- | --- |
| Emotional Stability | 0 | 0 | 0 | 0 | 0 | 0 |
| Extraversion | + | 0 | 0 | + | + | + |
| Openness | 0 | 0 | 0 | + | 0 | 0 |
| Altruism | 0 | 0 | 0 | 0 | + | 0 |
| Conscientiousness | + | + | 0 | 0 | + | 0 |

*Note.* “+” indicates a substantial positive correlation (i.e., above .30). 0 indicates a non-substantial correlation.

**Table A4**

Description of the Different Facets per Domain of the Business Attitudes Questionnaire

| **Facet** | **Low Level Description** | **High Level Description** |
| --- | --- | --- |
| **Emotional Stability** |  |  |
| Relaxed | Anxious or guilty in the event of failure, worried, lacking in calmness, nervous | Free from anxiety, maintains a calm attitude in the event of failure, calm and relaxed |
| Optimistic | Expects things to go wrong, worries about how things will turn out, pessimistic | Confident about the outcome of things, does not worry, remains cheerful |
| Stress-resistant | Sensitive to stress, copes poorly with tension and pressure, quickly affected by situations | Not very sensitive to stress, not particularly bothered by tension and pressure, not easily affected by situations |
| Decisive | Hesitates over decisions, needs time to reach conclusions | Makes decisions quickly, based on (in)sufficient data, draws firm conclusions |
| **Extraversion** |  |  |
| Leading | Lets others take the lead, gives away the initiative, doesn’t like giving instructions | Likes to lead, takes the initiative, gives others instructions |
| Communicative | Not fond of speaking, has trouble to keep the conversation going, is inarticulate | Is fond of speaking, keeps conversation going, is articulate |
| Persuasive | Poor salesperson, uncomfortable in negotiations, not convincing | Able to sell, comfortable in negotiations, convincing |
| Motivating | Uninspiring, lacks a motivating influence, leaves others to fend for themselves | Inspires others, has a motivating influence, inspires enthusiasm for the task |
| **Openness** |  |  |
| Abstract | Concrete, both feet on the ground, practical-minded | Theoretical, intellectually curious, likes complex, abstract things |
| Innovative | Lacks inventiveness and creativity, rarely thinks of new ways of seeing things | Is creative, generates new ideas and thinks of original ways of seeing things |
| Change-oriented | Prefers routine, needs security, prefers regularity to variety | Likes change, tries out new things, prefers variety to regularity |
| Open-minded | Does not see many possibilities, has trouble thinking up alternatives and options | Sees various possibilities, thinks up alternatives and options |
| **Altruism** |  |  |
| People-oriented | Enjoys being alone, is not very fond of company, is focused on him/herself, doesn’t need company | Enjoys group situations, is fond of company, is focused on others, seeks out company |

**Table A4 Continued**

| **Facet** | **Low Level Description** | **High Level Description** |
| --- | --- | --- |
| Cooperating | Rarely consults or involves others, does not seek cooperation, places own interests above those of the group | Consults and involves others, seeks cooperation, places group’s interests above his/her own |
| Helpful | Self-involved, lacks a helpful attitude, isn’t concerned about others, lacks considerateness | Helps when there are problems, gives advice, is considerate |
| Socially Confident | Finds it hard to establish contact, doesn’t always get along with people, unfriendly, unpleasant | Establishes contact easily, cheerful, gets along with people, friendly, pleasant, spontaneous |
| **Conscientiousness** |  |  |
| Organized | Does not work to a plan, sets insufficient deadlines, overlooks routine tasks | Plans carefully in the light of priorities, sets deadlines, keeps routine tasks in mind |
| Meticulous | Not very methodical or meticulous, has little eye for detail | Works methodically and meticulously, pays attention to details |
| Rational | Pays little attention to facts, relies on intuition, tends not to quantify, speaks or acts first and thinks afterwards | Sticks to the facts, evaluates and measures, quantifies, thinks twice before speaking or acting |
| Persevering | Loses heart quickly, gives up when facing opposition, rarely sees tasks through to a successful conclusion | Perseveres despite setback, keeps trying, persists in the face of opposition |
| **Professionalism** |  |  |
| Ambitious | Not very career-minded, unambitious, sets moderate objectives | Career-minded, ambitious, sets difficult objectives, aims high |
| Critical | Not very critical in his/her approach, accepts information or ideas from others without question | Examines information critically, identifies potential drawbacks and limitations |
| Results-oriented | Not very results-oriented, feels little need to achieve results, lacks competitiveness | Likes to achieve results, wants to stand out, is competitive |
| Strategic | Sets short-term objectives, looks at things from an operational or short-term perspective | Sets long-term objectives, looks at things from a strategic or long-term perspective |
| Autonomous | Adapts to the situation, adapts to the circumstances, does not show own approach or opinion | Influences the situation, marks the situation with his/her personality, has his/her own approach and opinion |

**Table A5**

Overview of the Coding Scheme for CSCPS, Including Categories, Sub-Categories and Items.

| **Category** | **Sub-category** | **Item** |
| --- | --- | --- |
| A: Establishing, constructing and maintaining shared knowledge and understanding | A1: Sharing knowledge and understanding of problems and solutions | Proposing appropriate solutions or introducing new appropriate information related to the problem  Talking about givens and constraints of a specific task  Building on others' ideas to improve solutions |
|  | A2: Establishing common ground | Asking for further clarification  Giving feedback on the understanding of what the other is saying and asking questions  Eliciting feedback from the one who is listening  Clarifying any information needs and responding to questions  Evoking turn-taking by means of explicit handovers  Repairing misunderstandings |
| B: Negotiating and coordinating for task completion and problem solving | B1: Responding to others' ideas or proposed solutions | Providing reasons to support a potential solution  Questioning, correcting or pointing out others' mistakes  Confirming to support a potential solution |
|  | B2: Monitoring execution | Talking about or discussing the results |
|  | B3: Time management | Monitoring time |
|  | B4: Technical coordination | Using the technical tools |
|  | B5: Discussing strategies | Discussing the general group strategies |
| C: Maintaining team function and organization | C1: Taking initiatives to advance collaboration processes | Asking if others have suggestions  Asking to take action before anyone on the team asks for help  Complimenting or encouraging others  Apologizing for one’s mistake(s).  Proposing to ask or asking for help outside of the group |
|  | C2: Coordinating task division | Defining (sub)tasks and talking about the adoption of these tasks |

##

# Appendix B

This appendix includes additional descriptive statistics for the questionnaire data used in this study. First, Table B1 presents the correlation coefficients between each of the BAQ domains and facets. Second, Table B2 presents the correlation coefficients between the TREO dimensions. Third, Table B3 presents descriptive statistics of the BAQ domains and facets. Lastly, Table B4 contains the descriptive statistics of the TREO dimensions.

**Table B1**

Correlations Between the BAQ Domains and Facets

|  | **ES** | **ES1** | **ES2** | **ES3** | **ES4** | **E** | **E1** | **E2** | **E3** | **E4** |
| --- | --- | --- | --- | --- | --- | --- | --- | --- | --- | --- |
| **Emotional Stability (ES)** | 1 | 0.81 *** | 0.63 *** | 0.83 *** | 0.65 *** | 0.38 | 0.26 | 0.23 | 0.41 | 0.4 |
| Relaxed (ES1) | 0.81 *** | 1 | 0.32 | 0.76 *** | 0.25 | -0.03 | -0.09 | -0.14 | 0.08 | 0.05 |
| Optimistic (ES2) | 0.63 *** | 0.32 * | 1 | 0.19 | 0.26 | 0.4 | 0.19 | 0.38 | 0.26 | 0.52 * |
| Stress resistant (ES3) | 0.83 *** | 0.76 *** | 0.19 | 1 | 0.5 * | 0.23 | 0.15 | 0.07 | 0.35 | 0.21 |
| Decisive (ES4) | 0.65 *** | 0.25 | 0.26 * | 0.5 *** | 1 | 0.56 ** | 0.56 ** | 0.37 | 0.55 ** | 0.39 |
| **Extraversion (E)** | 0.38 ** | -0.03 | 0.4 ** | 0.23 | 0.56 *** | 1 | 0.84 *** | 0.79 *** | 0.9 *** | 0.8 *** |
| Leading (E1) | 0.26 * | -0.09 | 0.19 | 0.15 | 0.56 *** | 0.84 *** | 1 | 0.49 * | 0.74 *** | 0.56 ** |
| Communicative (E2) | 0.23 | -0.14 | 0.38 ** | 0.07 | 0.37 ** | 0.79 *** | 0.49 *** | 1 | 0.63 *** | 0.5 * |
| Persuasive (E3) | 0.41 ** | 0.08 | 0.26 * | 0.35 ** | 0.55 *** | 0.9 *** | 0.74 *** | 0.63 *** | 1 | 0.65 *** |
| Motivating (E4) | 0.4 ** | 0.05 | 0.52 *** | 0.21 | 0.39 ** | 0.8 *** | 0.56 *** | 0.5 *** | 0.65 *** | 1 |
| **Openness (O)** | 0.48 *** | 0.44 *** | 0.25 | 0.41 ** | 0.31 * | 0.17 | 0.04 | 0.19 | 0.25 | 0.1 |
| Abstract (O1) | 0.19 | 0.25 | 0.01 | 0.21 | 0.1 | 0.04 | -0.11 | 0.1 | 0.16 | -0.01 |
| Innovative (O2) | 0.54 *** | 0.42 *** | 0.35 ** | 0.37 ** | 0.45 *** | 0.31 * | 0.2 | 0.28 * | 0.32 * | 0.25 |
| Change oriented (O3) | 0.6 *** | 0.58 *** | 0.26 * | 0.57 *** | 0.36 ** | 0.09 | 0.05 | 0.11 | 0.16 | -0.01 |
| Open minded (O4) | 0.3 * | 0.19 | 0.31 * | 0.23 | 0.12 | 0.15 | 0.06 | 0.14 | 0.16 | 0.14 |
| **Altruism (A)** | 0.22 | -0.05 | 0.43 *** | 0.05 | 0.18 | 0.49 *** | 0.34 ** | 0.47 *** | 0.31 * | 0.53 *** |
| People oriented (A1) | 0.2 | 0.1 | 0.32 * | 0.11 | 0.02 | 0.26 * | 0.2 | 0.24 | 0.16 | 0.26 * |
| Cooperating (A2) | 0.06 | -0.09 | 0.15 | 0.04 | 0.07 | 0.34 ** | 0.3 * | 0.21 | 0.22 | 0.4 ** |
| Helpful (A3) | 0.07 | -0.19 | 0.26 * | -0.06 | 0.19 | 0.28 * | 0.22 | 0.24 | 0.11 | 0.39 ** |
| Socially Confident (A4) | 0.29 * | -0.01 | 0.54 *** | 0.04 | 0.28 * | 0.6 *** | 0.32 * | 0.71 *** | 0.41 ** | 0.56 *** |
| **Conscientiousness (C)** | -0.19 | -0.36 ** | 0.03 | -0.29 * | 0.09 | 0.05 | 0.09 | 0.03 | -0.05 | 0.07 |
| Organised (C1) | -0.14 | -0.38 ** | 0.12 | -0.26 * | 0.15 | 0.22 | 0.3 * | 0.12 | 0.1 | 0.23 |
| Meticulous (C2) | -0.24 | -0.34 ** | 0.11 | -0.38 ** | -0.1 | 0.01 | -0.01 | 0.06 | -0.12 | 0.12 |
| Rational (C3) | -0.2 | -0.2 | -0.14 | -0.18 | -0.03 | -0.14 | -0.12 | -0.02 | -0.16 | -0.17 |
| Persevering (C4) | 0.09 | -0.04 | -0.06 | 0.07 | 0.37 ** | 0 | 0.1 | -0.12 | 0.07 | -0.05 |
| **Professionalism (P)** |  |  |  |  |  |  |  |  |  |  |
| Ambitious (P1) | 0.26 * | 0.19 | -0.14 | 0.33 * | 0.48 *** | 0.26 * | 0.37 ** | 0.07 | 0.29 * | 0.11 |
| Critical (P2) | 0.01 | -0.09 | -0.06 | 0.05 | 0.18 | 0.15 | 0.07 | 0.3 * | 0.19 | -0.08 |
| Result oriented (P3) | -0.04 | -0.14 | -0.08 | -0.01 | 0.15 | 0.29 * | 0.42 *** | 0.11 | 0.31 * | 0.11 |
| Strategic (P4) | 0.24 | 0.09 | 0.1 | 0.24 | 0.31 * | 0.32 * | 0.28 * | 0.26 * | 0.37 ** | 0.15 |
| Autonomous (P5) | 0.5 *** | 0.25 | 0.33 * | 0.4 ** | 0.52 *** | 0.42 *** | 0.31 * | 0.43 *** | 0.39 ** | 0.28 * |

*Note.* Personality domains are marked in bold. **p* < .05, ***p* < .01, ****p* < .001.

**Table B1 Continued**

|  | **O** | **O1** | **O2** | **O3** | **O4** | **A** | **A1** | **A2** | **A3** | **A4** |
| --- | --- | --- | --- | --- | --- | --- | --- | --- | --- | --- |
| **Emotional Stability (ES)** | 0.48 | 0.19 | 0.54 ** | 0.6 *** | 0.3 | 0.22 | 0.2 | 0.06 | 0.07 | 0.29 |
| Relaxed (ES1) | 0.44 | 0.25 | 0.42 | 0.58 *** | 0.19 | -0.05 | 0.1 | -0.09 | -0.19 | -0.01 |
| Optimistic (ES2) | 0.25 | 0.01 | 0.35 | 0.26 | 0.31 | 0.43 | 0.32 | 0.15 | 0.26 | 0.54 ** |
| Stress resistant (ES3) | 0.41 | 0.21 | 0.37 | 0.57 ** | 0.23 | 0.05 | 0.11 | 0.04 | -0.06 | 0.04 |
| Decisive (ES4) | 0.31 | 0.1 | 0.45 | 0.36 | 0.12 | 0.18 | 0.02 | 0.07 | 0.19 | 0.28 |
| **Extraversion (E)** | 0.17 | 0.04 | 0.31 | 0.09 | 0.15 | 0.49 * | 0.26 | 0.34 | 0.28 | 0.6 *** |
| Leading (E1) | 0.04 | -0.11 | 0.2 | 0.05 | 0.06 | 0.34 | 0.2 | 0.3 | 0.22 | 0.32 |
| Communicative (E2) | 0.19 | 0.1 | 0.28 | 0.11 | 0.14 | 0.47 | 0.24 | 0.21 | 0.24 | 0.71 *** |
| Persuasive (E3) | 0.25 | 0.16 | 0.32 | 0.16 | 0.16 | 0.31 | 0.16 | 0.22 | 0.11 | 0.41 |
| Motivating (E4) | 0.1 | -0.01 | 0.25 | -0.01 | 0.14 | 0.53 ** | 0.26 | 0.4 | 0.39 | 0.56 ** |
| **Openness (O)** | 1 | 0.83 *** | 0.86 *** | 0.74 *** | 0.76 *** | -0.17 | -0.29 | -0.21 | -0.08 | 0.06 |
| Abstract (O1) | 0.83 *** | 1 | 0.52 * | 0.43 | 0.52 * | -0.22 | -0.33 | -0.21 | -0.1 | -0.04 |
| Innovative (O2) | 0.86 *** | 0.52 *** | 1 | 0.64 *** | 0.66 *** | 0 | -0.18 | -0.06 | 0.02 | 0.2 |
| Change oriented (O3) | 0.74 *** | 0.43 *** | 0.64 *** | 1 | 0.39 | -0.08 | -0.07 | -0.09 | -0.14 | 0.03 |
| Open minded (O4) | 0.76 *** | 0.52 *** | 0.66 *** | 0.39 ** | 1 | -0.21 | -0.31 | -0.31 | -0.02 | 0.02 |
| **Altruism (A)** | -0.17 | -0.22 | 0 | -0.08 | -0.21 | 1 | 0.83 *** | 0.77 *** | 0.63 *** | 0.79 *** |
| People oriented (A1) | -0.29 * | -0.33 ** | -0.18 | -0.07 | -0.31 * | 0.83 *** | 1 | 0.68 *** | 0.27 | 0.51 * |
| Cooperating (A2) | -0.21 | -0.21 | -0.06 | -0.09 | -0.31 * | 0.77 *** | 0.68 *** | 1 | 0.29 | 0.37 |
| Helpful (A3) | -0.08 | -0.1 | 0.02 | -0.14 | -0.02 | 0.63 *** | 0.27 * | 0.29 * | 1 | 0.48 |
| Socially Confident (A4) | 0.06 | -0.04 | 0.2 | 0.03 | 0.02 | 0.79 *** | 0.51 *** | 0.37 ** | 0.48 *** | 1 |
| **Conscientiousness (C)** | -0.23 | -0.21 | -0.12 | -0.33 ** | -0.04 | 0.07 | -0.04 | -0.07 | 0.3 * | 0.07 |
| Organised (C1) | -0.36 ** | -0.38 ** | -0.21 | -0.34 ** | -0.16 | 0.2 | 0.12 | 0.06 | 0.32 * | 0.14 |
| Meticulous (C2) | -0.34 ** | -0.26 * | -0.23 | -0.46 *** | -0.16 | 0.18 | 0.06 | 0.01 | 0.31 * | 0.2 |
| Rational (C3) | 0.26 * | 0.27 * | 0.21 | 0 | 0.32 * | -0.28 * | -0.37 ** | -0.28 * | 0.04 | -0.18 |
| Persevering (C4) | -0.15 | -0.18 | -0.07 | -0.09 | -0.1 | 0.04 | 0.04 | -0.03 | 0.16 | -0.03 |
| **Professionalism (P)** |  |  |  |  |  |  |  |  |  |  |
| Ambitious (P1) | 0.27 * | 0.23 | 0.23 | 0.36 ** | 0 | -0.08 | -0.11 | -0.09 | 0.08 | -0.09 |
| Critical (P2) | 0.52 *** | 0.52 *** | 0.37 ** | 0.23 | 0.49 *** | -0.2 | -0.34 ** | -0.32 * | 0.04 | 0.03 |
| Result oriented (P3) | 0 | -0.03 | -0.02 | -0.03 | 0.12 | -0.05 | 0.05 | 0.03 | -0.1 | -0.14 |
| Strategic (P4) | 0.63 *** | 0.59 *** | 0.48 *** | 0.36 ** | 0.54 *** | -0.19 | -0.24 | -0.18 | -0.08 | -0.06 |
| Autonomous (P5) | 0.46 *** | 0.3 * | 0.51 *** | 0.34 ** | 0.35 ** | 0.11 | -0.03 | -0.13 | 0.23 | 0.27 * |

*Note.* Personality domains are marked in bold. **p* < .05, ***p* < .01, ****p* < .001.

**Table B1 Continued**

|  | **C** | **C1** | **C2** | **C3** | **C4** | **P** | **P1** | **P2** | **P3** | **P4** | **P5** |
| --- | --- | --- | --- | --- | --- | --- | --- | --- | --- | --- | --- |
| **Emotional Stability (ES)** | -0.19 | -0.14 | -0.24 | -0.2 | 0.09 |  | 0.26 | 0.01 | -0.04 | 0.24 | 0.5 * |
| Relaxed (ES1) | -0.36 | -0.38 | -0.34 | -0.2 | -0.04 |  | 0.19 | -0.09 | -0.14 | 0.09 | 0.25 |
| Optimistic (ES2) | 0.03 | 0.12 | 0.11 | -0.14 | -0.06 |  | -0.14 | -0.06 | -0.08 | 0.1 | 0.33 |
| Stress resistant (ES3) | -0.29 | -0.26 | -0.38 | -0.18 | 0.07 |  | 0.33 | 0.05 | -0.01 | 0.24 | 0.4 |
| Decisive (ES4) | 0.09 | 0.15 | -0.1 | -0.03 | 0.37 |  | 0.48 | 0.18 | 0.15 | 0.31 | 0.52 * |
| **Extraversion (E)** | 0.05 | 0.22 | 0.01 | -0.14 | 0 |  | 0.26 | 0.15 | 0.29 | 0.32 | 0.42 |
| Leading (E1) | 0.09 | 0.3 | -0.01 | -0.12 | 0.1 |  | 0.37 | 0.07 | 0.42 | 0.28 | 0.31 |
| Communicative (E2) | 0.03 | 0.12 | 0.06 | -0.02 | -0.12 |  | 0.07 | 0.3 | 0.11 | 0.26 | 0.43 |
| Persuasive (E3) | -0.05 | 0.1 | -0.12 | -0.16 | 0.07 |  | 0.29 | 0.19 | 0.31 | 0.37 | 0.39 |
| Motivating (E4) | 0.07 | 0.23 | 0.12 | -0.17 | -0.05 |  | 0.11 | -0.08 | 0.11 | 0.15 | 0.28 |
| **Openness (O)** | -0.23 | -0.36 | -0.34 | 0.26 | -0.15 |  | 0.27 | 0.52 * | 0 | 0.63 *** | 0.46 |
| Abstract (O1) | -0.21 | -0.38 | -0.26 | 0.27 | -0.18 |  | 0.23 | 0.52 * | -0.03 | 0.59 *** | 0.3 |
| Innovative (O2) | -0.12 | -0.21 | -0.23 | 0.21 | -0.07 |  | 0.23 | 0.37 | -0.02 | 0.48 | 0.51 * |
| Change oriented (O3) | -0.33 | -0.34 | -0.46 | 0 | -0.09 |  | 0.36 | 0.23 | -0.03 | 0.36 | 0.34 |
| Open minded (O4) | -0.04 | -0.16 | -0.16 | 0.32 | -0.1 |  | 0 | 0.49 * | 0.12 | 0.54 ** | 0.35 |
| **Altruism (A)** | 0.07 | 0.2 | 0.18 | -0.28 | 0.04 |  | -0.08 | -0.2 | -0.05 | -0.19 | 0.11 |
| People oriented (A1) | -0.04 | 0.12 | 0.06 | -0.37 | 0.04 |  | -0.11 | -0.34 | 0.05 | -0.24 | -0.03 |
| Cooperating (A2) | -0.07 | 0.06 | 0.01 | -0.28 | -0.03 |  | -0.09 | -0.32 | 0.03 | -0.18 | -0.13 |
| Helpful (A3) | 0.3 | 0.32 | 0.31 | 0.04 | 0.16 |  | 0.08 | 0.04 | -0.1 | -0.08 | 0.23 |
| Socially Confident (A4) | 0.07 | 0.14 | 0.2 | -0.18 | -0.03 |  | -0.09 | 0.03 | -0.14 | -0.06 | 0.27 |
| **Conscientiousness (C)** | 1 | 0.82 *** | 0.85 *** | 0.69 *** | 0.5 * |  | 0.1 | 0.28 | 0.31 | 0.09 | 0.28 |
| Organised (C1) | 0.82 *** | 1 | 0.61 *** | 0.34 | 0.31 |  | 0.17 | 0.02 | 0.35 | 0.02 | 0.19 |
| Meticulous (C2) | 0.85 *** | 0.61 *** | 1 | 0.49 * | 0.24 |  | -0.12 | 0.17 | 0.17 | -0.06 | 0.13 |
| Rational (C3) | 0.69 *** | 0.34 ** | 0.49 *** | 1 | 0.16 |  | 0.14 | 0.65 *** | 0.2 | 0.41 | 0.36 |
| Persevering (C4) | 0.5 *** | 0.31 * | 0.24 | 0.16 | 1 |  | 0.16 | -0.04 | 0.17 | -0.12 | 0.18 |
| **Professionalism (P)** |  |  |  |  |  |  |  |  |  |  |  |
| Ambitious (P1) | 0.1 | 0.17 | -0.12 | 0.14 | 0.16 |  | 1 | 0.29 | 0.41 | 0.4 | 0.37 |
| Critical (P2) | 0.28 * | 0.02 | 0.17 | 0.65 *** | -0.04 |  | 0.29 * | 1 | 0.25 | 0.65 *** | 0.5 * |
| Result oriented (P3) | 0.31 * | 0.35 ** | 0.17 | 0.2 | 0.17 |  | 0.41 ** | 0.25 | 1 | 0.36 | 0.18 |
| Strategic (P4) | 0.09 | 0.02 | -0.06 | 0.41 ** | -0.12 |  | 0.4 ** | 0.65 *** | 0.36 ** | 1 | 0.45 |
| Autonomous (P5) | 0.28 * | 0.19 | 0.13 | 0.36 ** | 0.18 |  | 0.37 ** | 0.5 *** | 0.18 | 0.45 *** | 1 |

*Note.* Personality domains are marked in bold. **p* < .05, ***p* < .01, ****p* < .001.

**Table B2**

Correlations Between the TREO Dimensions

|  | **Task-Oriented** | | **Change-Oriented** | | **Socio-Emotional** | |
| --- | --- | --- | --- | --- | --- | --- |
|  | **Organizer** | **Doer** | **Challenger** | **Innovator** | **Team Builder** | **Connector** |
| Organizer | 1 | 0.08 | 0.27 | 0.21 | 0.28 | 0.35 |
| Doer | 0.08 | 1 | 0.26 | 0.41 | 0.31 | 0.16 |
| Challenger | 0.27 * | 0.26 | 1 | 0.76 *** | 0.25 | 0.41 |
| Innovator | 0.21 | 0.41 ** | 0.76 *** | 1 | 0.32 | 0.37 |
| Team Builder | 0.28 * | 0.31 * | 0.25 | 0.32 * | 1 | 0.71 *** |
| Connector | 0.35 ** | 0.16 | 0.41 ** | 0.37 ** | 0.71 *** | 1 |

*Note.* **p* < .05, ***p* < .01, ****p* < .001.

**Table B3**

Descriptive Statistics of the BAQ Domains and Facets

|  | **M** | **SD** | **Med.** | **Min.** | **Max.** |
| --- | --- | --- | --- | --- | --- |
| **Emotional Stability** | 3.62 | 0.49 | 3.67 | 2.33 | 4.50 |
| Relaxed | 3.29 | 0.72 | 3.33 | 1.50 | 4.67 |
| Optimistic | 3.90 | 0.74 | 3.83 | 2.17 | 5.00 |
| Stress resistant | 3.52 | 0.64 | 3.58 | 2.17 | 4.83 |
| Decisive | 3.76 | 0.58 | 3.83 | 2.50 | 5.00 |
| **Extraversion** | 3.40 | 0.59 | 3.38 | 1.92 | 4.79 |
| Leading | 3.27 | 0.76 | 3.50 | 1.50 | 4.67 |
| Communicative | 3.37 | 0.73 | 3.25 | 2.17 | 4.83 |
| Persuasive | 3.47 | 0.67 | 3.50 | 1.67 | 5.00 |
| Motivating | 3.51 | 0.65 | 3.50 | 2.00 | 5.00 |
| **Openness** | 3.48 | 0.56 | 3.42 | 2.21 | 4.75 |
| Abstract | 2.93 | 0.97 | 3.17 | 1.00 | 5.00 |
| Innovative | 3.43 | 0.67 | 3.33 | 2.17 | 5.00 |
| Change oriented | 3.85 | 0.61 | 4.00 | 2.17 | 5.00 |
| Open minded | 3.73 | 0.54 | 3.83 | 2.67 | 4.83 |
| **Altruism** | 3.82 | 0.48 | 3.92 | 2.54 | 4.63 |
| People oriented | 3.37 | 0.69 | 3.42 | 2.00 | 5.00 |
| Cooperating | 3.79 | 0.61 | 3.83 | 1.50 | 5.00 |
| Helpful | 4.27 | 0.51 | 4.33 | 2.33 | 5.00 |
| Socially Confident | 3.85 | 0.70 | 4.00 | 1.67 | 5.00 |
| **Conscientiousness** | 3.72 | 0.49 | 3.75 | 2.46 | 4.58 |
| Organised | 3.48 | 0.76 | 3.33 | 1.67 | 5.00 |
| Meticulous | 3.55 | 0.78 | 3.50 | 1.83 | 5.00 |
| Rational | 3.78 | 0.64 | 4.00 | 2.33 | 5.00 |
| Persevering | 4.05 | 0.46 | 4.00 | 2.50 | 4.83 |
| **Professionalism** |  |  |  |  |  |
| Ambitious | 3.46 | 0.66 | 3.50 | 2.17 | 5.00 |
| Critical | 4.07 | 0.62 | 4.00 | 2.33 | 5.00 |
| Result oriented | 3.63 | 0.55 | 3.67 | 2.50 | 4.83 |
| Strategic | 3.63 | 0.66 | 3.67 | 1.17 | 5.00 |
| Autonomous | 3.89 | 0.48 | 3.83 | 3.00 | 5.00 |

**Table B4**

Descriptive Statistics of the TREO Dimensions

|  | **M** | **SD** | **Med.** | **Min.** | **Max.** |
| --- | --- | --- | --- | --- | --- |
| Organizer | 3.85 | 0.69 | 4.00 | 2.00 | 5.00 |
| Doer | 3.35 | 0.72 | 3.33 | 2.00 | 4.67 |
| Challenger | 3.06 | 0.70 | 3.00 | 1.50 | 4.50 |
| Innovator | 3.48 | 0.73 | 3.67 | 1.33 | 4.67 |
| Team Builder | 3.37 | 0.83 | 3.33 | 1.00 | 5.00 |
| Connector | 3.85 | 0.69 | 4.00 | 2.00 | 5.00 |

# Appendix C

This appendix includes a summarized overview of the results of the correlation analyses for Q1 (see Table C1) compared to the hypotheses.

**Table C1**

Overview of the Hypotheses and Results of Q1

|  | **Organizer** | **Doer** | **Challenger** | **Innovator** | **Team builder** | **Connector** |
| --- | --- | --- | --- | --- | --- | --- |
| Emotional Stability | 0  0 | 0  0 | 0  0 | 0  0 | 0  0 | 0  0 |
| Extraversion | +  + | 0  0 | +  0 | 0  + | +  + | +  + |
| Openness | 0  0 | 0  0 | +  0 | +  + | 0  0 | 0  0 |
| Altruism | +  0 | 0  0 | 0  0 | 0  0 | +  + | +  0 |
| Conscien-tiousness | 0  + | 0  + | 0  0 | 0  0 | 0  + | 0  0 |

*Note.* Results from this study are shown in the top left corner of each cell. The hypotheses as referred to earlier, are shown in the bottom right corner of each cell. “+” indicates a substantial positive correlation (i.e., of above .30). 0 indicates a non-substantial correlation.
